# Increased Susceptibility to Obesity and Glucose Intolerance in Adult Female Rats Programmed by High-Protein Diet during Gestation, But Not during Lactation

**DOI:** 10.3390/nu12020315

**Published:** 2020-01-25

**Authors:** Caroline Desclée de Maredsous, Gabrielle Carlin, Annemarie Oosting, Corine Delteil, Dalila Azzout-Marniche, Catherine Chaumontet, François Blachier, Pierre Barbillon, Tristan Mary-Huard, Daniel Tomé, Raish Oozeer, Anne-Marie Davila

**Affiliations:** 1 Université Paris-Saclay, AgroParisTech, INRAE, UMR PNCA, 75005 Paris, France; caroline.desclee@danone.com (C.D.d.M.); carlin.g@orange.fr (G.C.); corine.delteil@agroparistech.fr (C.D.); dalila.azzout_marniche@agroparistech.fr (D.A.-M.); catherine.chaumontet@agroparistech.fr (C.C.); francois.blachier@agroparistech.fr (F.B.); daniel.tome@agroparistech.fr (D.T.); 2 Danone Nutricia Research, 3584 CT Utrecht, The Netherlands; annemarie.oosting@danone.com (A.O.); raish.oozeer@danone.com (R.O.); 3 Université Paris-Saclay, AgroParisTech, INRAE, UMR MIA-Paris, 75005 Paris, France; pierre.barbillon@agroparistech.fr (P.B.); tristan.mary-huard@agroparistech.fr (T.M.-H.)

**Keywords:** high-protein, perinatal, obesity risk

## Abstract

Fetal and early postnatal nutritional environments contribute to lifelong health. High-protein (HP) intake in early life can increase obesity risk in response to specific feeding conditions after weaning. This study investigated the effects of a maternal HP diet during pregnancy and/or lactation on the metabolic health of offspring. Three groups of dams received a normal-protein (NP, 20E% proteins) diet during gestation and lactation (Control group), an HP diet (55E% proteins) during gestation (HPgest group), or an HP diet during lactation (HPlact group). From weaning until 10 weeks, female pups were exposed to the NP, the HP or the western (W) diet. HPgest pups had more adipocytes (*p* = 0.009), more subcutaneous adipose tissue (*p* = 0.04) and increased expression of genes involved in liver fatty acid synthesis at 10 weeks (*p* < 0.05). HPgest rats also showed higher food intake and adiposity under the W diet compared to the Control and HPlact rats (*p* ≤ 0.04). The post-weaning HP diet reduced weight (*p* < 0.0001), food intake (*p* < 0.0001), adiposity (*p* < 0.0001) and glucose tolerance (*p* < 0.0001) compared to the NP and W diets; this effect was enhanced in the HPgest group (*p* = 0.04). These results show that a maternal HP diet during gestation, but not lactation, leads to a higher susceptibility to obesity and glucose intolerance in female offspring.

## 1. Introduction

Early life environment is recognized as an important programming factor for health in later life [1], and inadequate nutrition during critical periods of development results in increased susceptibility to obesity and metabolic disease [2]. Infancy is a critical developmental period [3] and postnatal growth trajectories are predictive for body mass index (BMI) in adulthood [4]. It has been suggested that differences in obesity-risk between formula and breastfed infants [5] are caused by accelerated growth of formula fed infants [6] due to higher protein intakes [7].

The protein content of maternal diet during pregnancy and/or lactation also influences offspring development. A protein restriction during gestation increases the risk of overweight and food control disorders in adult offspring [8,9,10,11]. High-protein (HP) intakes during pregnancy have shown inconsistent effects on birth outcomes [12,13,14] but a prospective cohort study demonstrated that the BMI of 20-year-old women was positively correlated with her mother’s animal protein intake during pregnancy [15]. Animal studies concerning HP diets during perinatal life generated conflicting results ([16,17] vs. [18,19]). However, in previous work [20] rat female offspring from dams fed a HP diet during gestation and allowed to self-select macronutrient after weaning increased their fat and protein intake at the expense of carbohydrate intake and had decreased insulin sensitivity and higher adiposity.

The aim of the present study was to investigate, in rats, whether a maternal HP diet during gestation or lactation increases the sensitivity of the offspring to obesity and metabolic dysfunctions in response to specific challenging diet after weaning including obesogenic diet. For this purpose, pups from mother fed a HP diet during gestation or lactation received a normal protein (NP) diet, an HP diet or a “western” (W) diet after weaning and were further characterized for susceptibility to obesity and glucose tolerance in adulthood.

## 2. Materials and Methods

### 2.1. Diets

Three different diets were used. The NP diet was composed of 20% energy (20E%) from proteins, 70E% from carbohydrates and 10E% from plant lipids (soybean oil). The HP diet contained 55E% from proteins and 35E% from carbohydrates with the same 10E% from lipids. The W diet had 20E% from proteins and 38E% from carbohydrates, including 19E% from sucrose and 42E% from animal lipids (lard). Both the NP and HP diet were isoenergetic with 3.5 kCal/g, and the W diet was higher in energy with 4.5 kCal/g (Supplementary Materials Table S1).

### 2.2. Experimental Design

**Figure 1:**
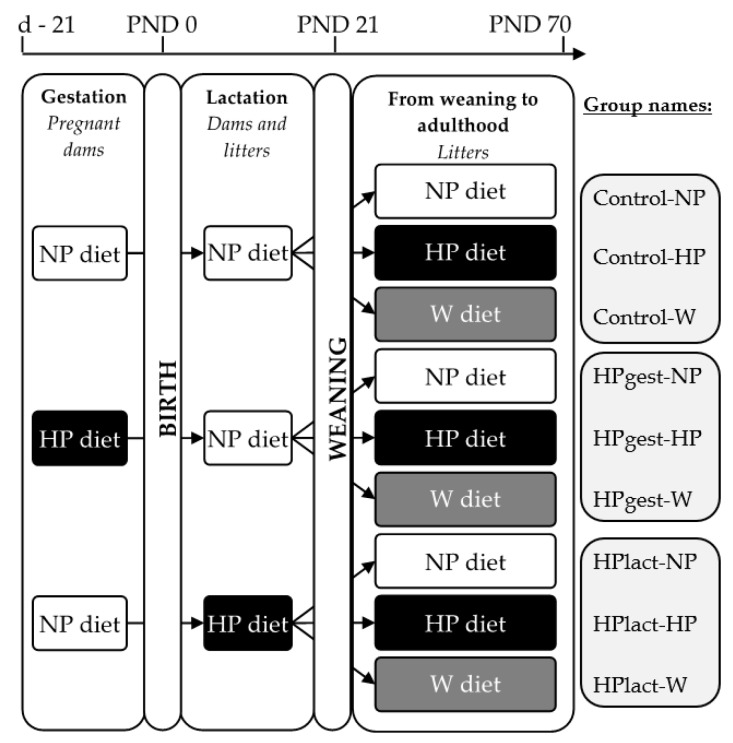
Experimental design. d: day, HP: high-protein, NP: control, PND: post-natal day, W: western.

The protocol was approved by the local animal ethical committee (COMETHEA, Jouy-en-Josas, France, Nos. DAP14_05). Eighteen female Wistar rats (Harlan, France) were maintained under controlled conditions (22 °C, 12:12 h dark-light cycle; lights on at 08:00) with free access to the diet and water. The females were randomly assigned to 3 different groups (6 dams/group): Control group, HPgest group and HPlact group (Figure 1). After one week of habituation (including 3–4 days of exposure to both chow and experimental diet), females were mated. From the day of mating, the HPgest group received the HP diet, and the Control and HPlact groups received the NP diet. At birth, litters were standardized to 8 pups giving priority to females. Control and HPgest groups received the NP diet, and the HPlact group started on the HP diet. At weaning, females of each litter were split into 3 groups and exposed to the NP, HP or W diet (Figure 1). Each pup was weighed daily from birth to 10 weeks. After weaning, food intake was monitored 3 times per week by weighing. At 10 weeks, after a night of ad libitum feeding, female pups were terminally anesthetized with pentobarbital sodium. Peri-ovarian adipose tissue, liver and hypothalamus were sampled, snap-frozen in liquid nitrogen and stored at −80 °C.

### 2.3. Body Composition Measurement by Magnetic Resonance Imaging (MRI)

The body composition of the pups was measured by MRI at weaning and at 9 weeks old. Images were acquired with a 4.7T Bruker Biospec system (running Paravision 5) using a Bruker 70 mm i.d. tunable quadrature RF resonator. The female pups were anesthetized with isoflurane in oxygen-supplemented air. Breathing rate was monitored, and body temperature was maintained at 36–38 °C with a water-heated bed. A TurboRARE-3D sequence was used to acquire fat-sensitive T2-weighted images (TR/TE = 750/42 ms, FOV 9 × 5 × 4 cm, resolution 520 × 521 × 519 µm at weaning and TR/TE = 750/73 ms, FOV 18 × 7 × 5.5 cm, resolution 522 × 522 × 524 µm at 9 weeks). Images were registered, then fat pads segmented semi-automatically (by fuzzy c-means) in MIPAV 4.3.0. Adipose volume was converted to grams of fat mass (FM) on the assumption of a density of 0.9 g cm^−3^ as previously described [20].

### 2.4. Oral Glucose Tolerance Test (OGTT)

OGTT was performed 3 days before sacrifice. After overnight fasting, a 400 µL blood sample was drawn by means of tail vein puncture. Subsequently, animals received 1 g/kg glucose by oral gavage. An amount of 150 µL blood samples were drawn at 15, 30, 60 and 120 min after glucose administration. Glucose was immediately assessed by applying a drop of blood to a test strip and then measuring by a glucose meter (Accu-Check Go, Roche Diagnostic, France). In addition, blood was collected in EDTA coated tubes, centrifuged at 4 °C, 3000× *g* for 10 min, and plasma was stored at −20 °C for further analysis. Plasma insulin was measured by ELISA, according to the manufacturer’s instructions (10-1250-01, Mercodia, Uppsala, Sweden).

### 2.5. Adipose Tissue Cellularity

Cellularity analysis was performed in peri-ovarian adipose tissue as previously described [21]. Approximately 40 mg of adipose tissue was digested by collagenase at 37 °C for 15 min. The sample was subsequently placed on a heated microscope slide and images of isolated adipocytes were taken with a camera-equipped microscope (Axioimager.Z1 and Axiocam MRc, Zeiss, Oberkochen, Germany). The diameter of at least 1000 cells was assessed by image analysis using ImageJ software (Bethesda, Maryland, USA) and then perimeter was calculated, allowing the calculation of the mean adipocyte weight (density of triglycerides = 0.92). Fat cell number was estimated by dividing the entire peri-ovarian adipose tissue lipid content by the mean adipocyte weight. Approximately 100 mg of the same adipose tissue was sampled and stored at −20 °C for lipid measurement. Lipid extraction was performed according to the method of Folch et al. [21] using dichloromethane-methanol (2:1, *v*/*v*).

### 2.6. Liver and Plasma Analyses

Liver triglycerides were measured as previously described [22]. Approximately 80 mg of the same liver lobe was weighed and homogenized in 1 mL buffer (150 mM NaCl, 10 mM tris, 0.1% triton), and triglycerides were measured by colorimetric enzymatic assay (TR210, triglycerides, Randox, Antrim, United Kingdom) in a 10 µL sample. Fasting plasma leptin, glucagon, glucagon-like peptide 1 (GLP-1) and peptide YY (PYY) were measured with a luminex assay (RMHMAG-84K-04, Rat Metabolic Hormone Magnetic Bead Multiplex Assay, Merk-Millipore, Fontenay-sous-bois, France). Samples, standards and quality control were prepared according to the manufacturers protocol, and fluorescence was measured with a Luminex 100 reader (Luminex 100 analyzer, Austin, TX, USA). Triglycerides, cholesterol, high-density lipoprotein (HDL) cholesterol, non-esterified fatty acids (NEFA), urea and hydroxybutyrate levels were measured using an Olympus AU 400 automatic chemical analyzer. Plasma insulin-like growth factor-1 (IGF-1) was measured by ELISA. An amount of 50 µL of 1000-fold diluted plasma samples were prepared and measured according to the manufacturer’s protocol (Mouse/Rat IGF-I quantikine ELISA MG100, R&D System, Lille, France).

### 2.7. Real-Time Quantitative PCR

Total RNA was extracted from the liver, adipose tissue and the hypothalamus using Trizol Reagents (Invitrogen). RNAs were quantified using NanoDrop (Isogen, Maarssen, The Netherlands). The synthesis of cDNA was performed on 0.4 µg of total RNA with a reverse transcription kit (4368813, Applied Biosystems, Foster City, CA, USA). Real-time PCR was performed using the SYBR green fast Reagent PCR master mix (Applied Biosystems) on a StepOnePlusTM real-time PCR system (Applied Biosystems). Primers are presented in Supplementary Materials Table S2. Ribosomal 18S was used as housekeeping gene to account for variations in the initial quantities of cDNA, and inter-plate variations were corrected using RT calibrator. Gene expression was calculated using the formula: 2^−ΔΔ*C*t^ with ΔΔ*C*_t_ = (*C*_t_ sample gene of interest–*C*_t_ sample 18S) − (*C*_t_ reference sample gene of interest–*C*_t_ reference sample 18S) with Control-NP group as the reference group.

### 2.8. Statistical Analysis

Group size calculation was performed with the PROC power function based on similar previous studies [20,23]. Two mixed models were used to analyze the data. Model 1 was used to analyze parameters that were measured only once, whereas Model 2 was used to analyze parameters that were measured repeatedly. Both Models 1 and 2 account for the (fixed) effects of gestation, lactation and post-weaning diets, as well as their interactions. A random rat dam effect was included to account for correlations among pups from the same dam. Model 2 also includes a time effect and interactions between time and diets. A random pup effect was included to account for correlations between repeated measurements from the same pup. Pairwise comparisons were adjusted for multiple comparisons using a Tukey post hoc analysis in Model 1 and using Bonferroni’s post hoc analysis in Model 2. All statistical analyses were performed using SAS 9.1 (SAS Institute, Cary, NC, USA). Differences between groups were considered significant at *p* < 0.05.

## 3. Results

### 3.1. Body Weight and Food Intake

**Figure 2:**
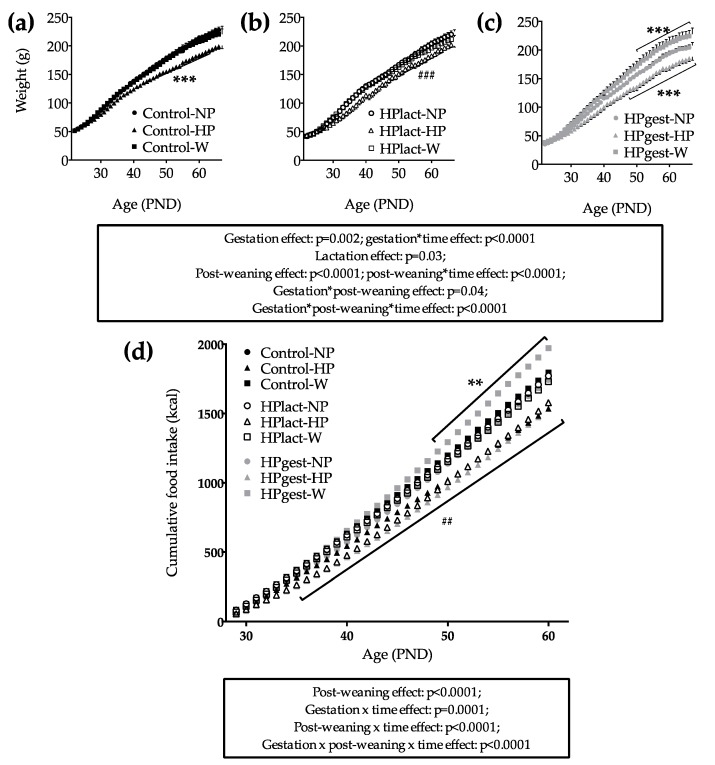
Body weight of the female rat pups of the Control group (**a**), the HPlact group (**b**) and the HPgest group (**c**) from PND21 to PND68 and cumulated food intake of the female rat pups after weaning (**d**) Data are presented as means ± SEM (*n* = 8). Effects of diets, time and their interactions were tested within mixed Model 2. *** indicates that the group is significantly different from the others of the graph, ### means that the group is significantly different from the HPlact-NP group. ## Post-weaning HP diet groups are significantly different than the others. ** HPgest-W is significantly different of HPgest-NP.

Gestation diet had no significant effect on the food intake of the dams during the last week of gestation (Supplementary Materials Figure S1). Birth weight was not affected by gestation diet (5.5 ± 0.07 g for NP and 5.3 ± 0.09 g for HP, *p* = 0.44). Cumulative food intake of dams during lactation (PND0 to PND19) was significantly lower in HPgest and HPlact groups (Supplementary Materials Figure S1). At the end of lactation, offspring body weight was significantly lower in the HPlact group and in the HPgest group compared to the control group (gestation effect: *p* < 0.0001, lactation effect: *p* = 0.0003, Supplementary Materials Table S3). From post-natal day (PND) 21 to PND68, pups fed with the post-weaning HP diet had a lower body weight than others (*p* < 0.0001, Figure 2). Moreover, the HPgest-W group gained more weight than the HPgest-NP and HPgest-HP groups (gestation × post-weaning interaction effect: *p* = 0.03, Figure 2c).

Cumulative food intake was significantly decreased due to the post-weaning HP diet (*p* < 0.0001, Figure 2d). Moreover, food intake was only increased in the HPgest group that was exposed to the post-weaning W diet (gestation × post-weaning interaction effect: *p* < 0.0001, Figure 2d).

### 3.2. Adiposity and Adipocyte Size

At the end of lactation, the HPlact pups showed lower total, visceral and subcutaneous fat mass (FM), both in absolute and relative to body weight values compared to Control pups (Supplementary Materials Table S3). The HPgest pups had a lower absolute total FM and a lower relative visceral FM compared to Control pups (visceral FM in % of body weight: 2.2 ± 0.07% for Control pups, 1.66 ± 0.06% for HPlact pups, 1.68 ± 0.06% for HPgest pups; gestation diet effect: *p* = 0.02, Supplementary Materials Table S3), whereas relative total and subcutaneous FM were not significantly affected by gestation diet.

At PND 65, pups receiving the post-weaning HP diet had less total, visceral and subcutaneous FM (post-weaning effect: *p* < 0.0001, *p* < 0.0001 and *p* < 0.005 for total, visceral and subcutaneous FM respectively, Table 1). The amount of visceral adipose tissue was increased only in the HPgest-W group (gestation × post weaning interaction effect: *p* = 0.03, Table 1). Subcutaneous FM was increased in pups of the HPgest group (*p* = 0.04, Table 1).

**Figure 3:**
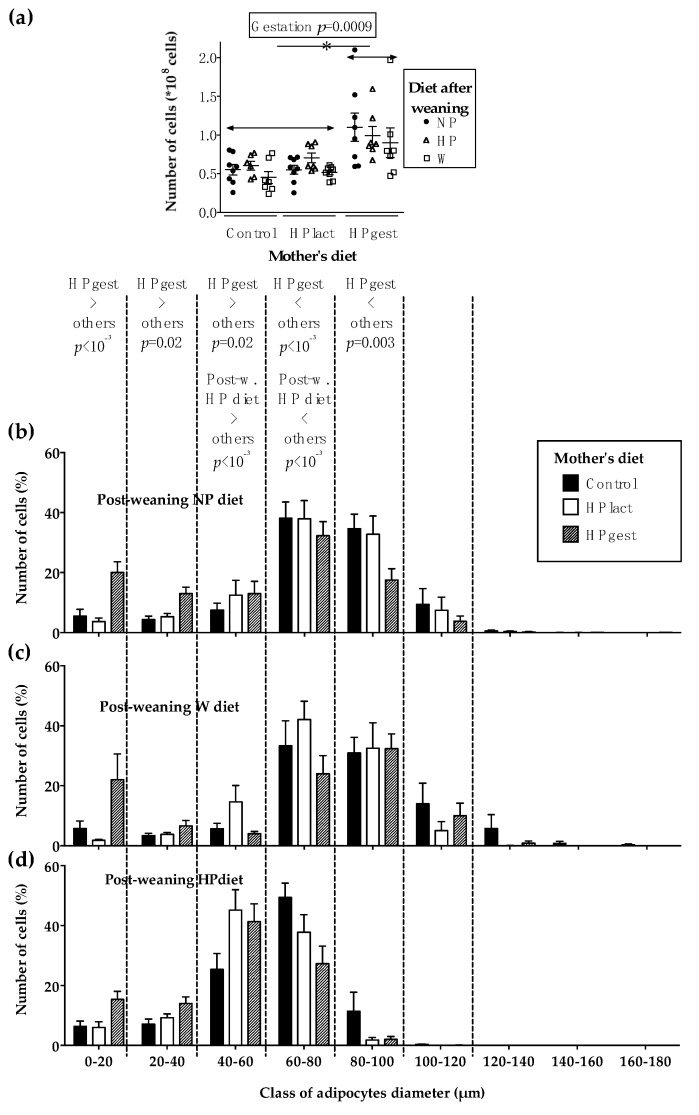
Adipocytes in peri-ovarian adipose tissue of female rat pups at PND70. Number of adipocytes in total peri-ovarian adipose tissue (**a**) and peri-ovarian adipocyte size of pups receiving the post-weaning NP diet (**b**), W diet (**c**) and HP diet (**d**). Data are represented as means ± SEM with individual values for (a), (*n* = 8). Effects of diets, time and their interactions were tested within mixed Model 1.

The size and number of adipocytes in the peri-ovarian fat depot were influenced by the gestation diet: the HPgest group had a higher number of adipocytes (gestation effect: *p* < 0.001, Figure 3a), with a higher percentage of small-sized adipocytes, and a lower percentage of medium-sized adipocytes (Figure 3b–d). The HP diet after weaning resulted in smaller adipocytes but did not affect the adipocyte number (Figure 3a–d).

### 3.3. Liver Fat and Plasma Lipids, Metabolites, Leptin and Gut Peptides

Liver triglyceride concentrations were increased due to the post-weaning W diet (*p* < 0.0001, Table 1). The post-weaning HP diet decreased plasma triglycerides (*p* = 0.03), but increased total and HDL cholesterol (*p* = 0.01 and *p* = 0.007, Table 1). There was no significant effect of the diets on the HDL/total cholesterol ratio. NEFA plasma concentrations were lower in the HPgest group (*p* = 0.04). Plasma urea was higher due to the post-weaning HP diet (*p* = 0.04, Supplementary Materials Table S4), whereas hydroxybutyrate, leptin and ratio of leptin to adiposity were lower (*p* < 0.0001, *p* < 0.0001 and *p* = 0.008 respectively, Supplementary Materials Table S4). At 10 weeks of age, plasma IGF-1 and fasted plasma peptide YY (PYY) did not differ between groups, but glucagon-like peptide 1 (GLP-1) was significantly increased due to the HP diet after weaning (post-weaning effect: *p* = 0.03, Supplementary Materials Table S4).

### 3.4. Glucose Homeostasis

Fasting blood glucose was increased in pups that received the post-weaning HP diet (*p* < 0.0001), but fasting plasma insulin and the HOMA-IR index did not differ between groups (Table 1). The HP diet during gestation enhanced the effect of the post-weaning diet on blood glucose with the HPgest-HP group having the highest fasting blood glucose (gestation × post weaning interaction effect: *p* = 0.02, Table 1). Fasting plasma glucagon was significantly higher in pups of the HPgest group (*p* = 0.02).

**Figure 4:**
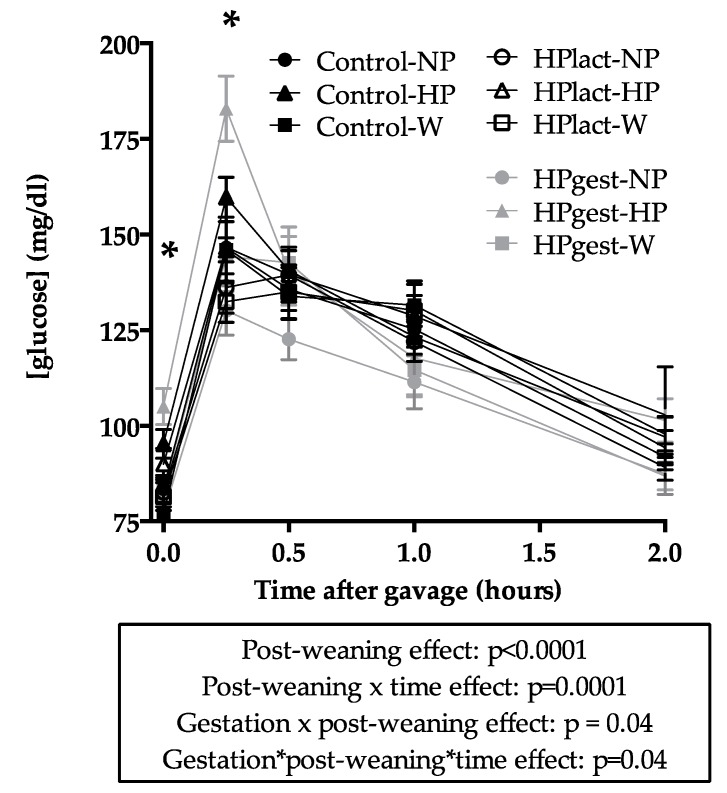
Blood glucose during an oral glucose tolerance test on female rat pups at PND68. Data are represented as means ± SEM, (*n* = 8). Effects of diets, time and their interactions were tested within mixed Model 2. * post-weaning HP diet groups significantly different than the others.

During the OGTT, pups that received the HP diet after weaning had higher blood glucose levels than the others (post-weaning effect and post-weaning × time interaction effect: *p* ≤ 0.0001, Figure 4). This effect was also amplified by the HP diet during gestation with a higher glucose peak for the HPgest-HP group (gestation × post-weaning and gestation × post-weaning × time interaction effects: *p* = 0.04 for both). During the OGTT, insulin response did not differ between groups (Supplementary Materials Figure S2).

### 3.5. Gene Expression

In the liver, fatty-acid synthase (FAS), acetyl-CoA carboxylase (ACC) and stearoyl coenzym-a desaturase 1 (Scd1) gene expressions were decreased due to the post-weaning W diet (*p* < 0.0001), and were increased in the HPgest group (gestation effect: *p* = 0.002, *p* = 0.006 and *p* = 0.006 respectively, Table 2). Liver glycerol-3-phosphate acyltransferase (GPAT) gene expression was also decreased in the pups exposed to the post-weaning W diet. In contrast, the expression of genes encoding for liver microsomal triglyceride transfer protein (MTTP), diglyceride acyltransferase (DGAT), carnitine palmitoyltransférase 1 (CPT1) and acyl-CoA oxydase 1 (Acox) were not significantly affected by the diets.

Liver L-pyruvate kinase (LPK) gene expression was increased by the HP diet during gestation (*p* = 0.049) and was lowered in pups exposed to both post-weaning HP and W diets compared to the NP diet (*p* < 0.0001). The post-weaning HP diet pups had a lower gene expression of liver glucokinase (GK) compared to the NP group. Liver glucose-6-phosphatase (G6PC1) gene expression was not significantly different between groups, but the liver phosphoenolpyruvate carboxykinase (PEPCK) gene expression was higher in pups that received the post-weaning HP diet (*p* = 0.007, Table 2).

In adipose tissue, FAS and ACC gene expression were decreased due to post-weaning W, whereas lipoprotein lipase (LPL), peroxisome proliferator-activated receptor γ (PPARγ) and IGF-1 receptor gene expressions were not significantly modified by diets (Supplementary Materials Table S5).

In the hypothalamus, expression of pro-opiomelanocortin (POMC) and cocaine- and amphetamine-regulated transcript (CART) were significantly decreased in the HPlact group (Supplementary Materials Table S6). The expression level of corticotrophin-releasing factor (CRF) was significantly higher in the HPgest group and the expression of neuropeptide Y (NPY) was increased in pups receiving the HP diet after weaning compared to the W diet (post weaning effect: *p* = 0.03, Supplementary Materials Table S6).

## 4. Discussion

This study explored the consequences of an HP diet exposure during gestation or lactation on later life adiposity and metabolic phenotype following exposure to post-weaning diet challenges. It is important to note that HP diet effects are an association of the effects of a diet with a high proportion of proteins and a low proportion of carbohydrates. The HP diet during gestation, but not during lactation, predisposed offspring to adult obesity and reduced glucose tolerance. Pups of dams that received the HP diet during gestation had more adipocytes, more subcutaneous adipose tissue and a potential increased liver fatty acid synthesis, as indicated by up-regulation of ACC, FAS and Sdc1 gene expression. A western diet challenge after weaning enhanced the adverse effects of the HP diet during gestation, resulting in a higher body weight gain, food intake and visceral FM compared to the NP diet after weaning. In contrast to the HP diet during gestation, the post-weaning HP diet induced direct effects like lower body weight gain, food intake and adiposity compared to the post-weaning NP diet, but also decreased glucose tolerance. The gestational HP diet followed by the post-weaning HP diet amplified this negative effect on glucose tolerance. Consistently with previous findings [24], the HP diet during lactation had almost no effect on pups’ later life metabolic health in this rat model.

The present results demonstrate that when the HP diet during gestation was combined with the W diet after weaning, the amount of visceral fat was increased, like what was found when pups were allowed to self-select a higher fat intake, even if associated with a higher intake of proteins and a lower intake of carbohydrates during the post-weaning period [20]. Furthermore, this study shows that prenatal exposure to the HP diet induced a higher fat mass and lipid storage capacity in pups. Regardless of the pup diet after weaning, an HP diet during gestation increased the number of adipocytes in the offspring peri-ovarian adipose tissue. The number of adipocytes is known to be set during childhood and adolescence and to remain mostly unchanged afterwards [25]. A higher number of adipocytes can directly lead to an increased risk of obesity [25]. The higher fat mass due to the gestational HP diet was associated with an increased expression of hepatic genes involved in the de novo fatty acid synthesis. Although adipose tissue development occurs after birth in rats [26], programming of adipose tissue can take place during fetal life [2,27]. For example, the programming of adipose tissue of rat pups exposed to in utero under-nutrition or maternal obesity seems associated with early induction of the adipogenic transcription factor PPARγ, whose activation leads to adipocyte differentiation [28]. In this study, the gene expression of PPARγ and IGF-1-receptor at 10 weeks of age was not affected by the experimental diets, but this does not exclude differential expression of PPARγ in early life due to the HP diet during gestation. Effects of the prenatal environment on adult hepatic lipid metabolism have been already described in a model of gestational protein restriction [29], maternal obesity [30] and maternal insulin resistance. Upregulation of fatty acid synthesis is often associated with hepatic steatosis [30,31], but liver triglyceride levels were not increased in our study. In addition, gene expression of CPT1 and Acox did not differ between groups, indicating that fatty acid oxidation was not affected by diets. In addition, the gene expression of the MTTP fatty-acid transporter was not different between groups, suggesting that fatty acids are exported to adipose tissue instead of being stored in the liver as ectopic fat.

This model shows a lack of long-term effects of the lactation HP diet on offspring metabolic health. The HPlact group pups had lower weight gain during lactation and a lower fat mass at 3 weeks. This could be a consequence of dams’ food intake and also of pups’ direct intake of the HP diet, but these effects are not sustainable after weaning. The only described HP diet long-term effect during lactation is a decreased gene expression of anorexigenic peptides POMC and CART at 10 weeks [32], but without association with lower food intake. This is not in line with the “early protein hypothesis”, which states that HP intake after birth leads to an increased obesity risk [33]. This can be related to the present rat model where the early HP intervention after birth did not directly target the pup but the mother, and it could be suggested that there were only small effects on milk composition. The direct HP diet exposure of pups started around the age of 12 to 15 days, when pups start to eat the mother’s diet, which may not be the right time window to confirm the “early protein hypothesis”.

The HP diet during gestation predisposed the pups to obesity, without inducing small birth weight. This trait was especially observed when pups were fed an obesogenic post-weaning diet (NP, containing high level of sucrose and animal fat) as already showed with the post-weaning DSS option, leading to HP/high-energy/high-fat intake [20]. Even if we did not observe an increased growth in HPgest offspring at the end of lactation, maternal diet during gestation may have impacted milk composition (as hormone or micronutrients) and production thereafter [34,35]. In addition it is important to note that HP diet effects are an association of the effects of a diet with a high proportion of proteins and a low proportion of carbohydrates. In contrast, when preceded by an NP diet with a lower proportion of proteins and a high proportion of carbohydrates during gestation and lactation, the W diet had limited effects. The W diet consumption increased triglyceride concentration in the liver, which is a well established effect of obesogenic diets [36]. The gene expressions of FAS and ACC in liver and adipose tissue, and Scd1 in the liver were also decreased in pups receiving the W diet, suggesting a down-regulation of fatty-acid synthesis. As tissues were sampled in a fed state, this down-regulation could be related to the higher fat content of the diet that lowers the need for de novo fatty-acid production. The HP diet during gestation increased post-weaning food intake in response to the W diet, whereas no difference was observed in response to DSS option (separate macronutrient options) in a previous study [20], suggesting that a diet challenge may be required to affect energy intake. Food intake is controlled by brain regions responsible for energy homeostasis and those involved in cognitive/reward homeostasis [37]. Both can be programmed by early life environment [38,39]. However, we did not find any programming effect of the gestation diet on gene expression of POMC, CART, NPY, AgRP, MC4-R, CRF, Y2R or Y5R in rat pup hypothalami. This could be related to the tissue sampling performed after a night of ad libitum feeding. Our findings concerning the hypothalamus need to be confirmed, but the reward system could also be the key regulator of disturbed food intake regulation in the HPgest group pups, and their increased food intake in response to the highly palatable W diet.

The present study also showed metabolic effects of the post-weaning HP diet, which is in accordance with previous findings in adults [24,40,41,42,43]. Young animals seem more responsive to the HP diet than adults; pups fed the HP diet after weaning had a 10% lower weight than pups fed the NP diet, whereas the weight effect of HP diets on adults can be around 4–6% [40,44]. In spite of similar liver triglyceride concentrations in all groups, the post-weaning HP diet decreased liver expression of genes coding for enzymes of the de novo fatty-acids synthesis (FAS and ACC). This is in line with previous findings showing that exposing adult rats to an HP diet reduced hepatic fatty acid synthesis [43,45]. The post-weaning HP diet also resulted in smaller adipocytes, associated with reduced adiposity [44]. Unlike previous studies, this study showed that fasting β-hydroxybutyrate plasma concentration was decreased by the post-weaning HP diet, but this could be related to measurements performed in the fasted state [43].

The post-weaning HP diet was also associated with higher blood glucose levels during the OGTT, a potential early sign of glucose intolerance, confirming the effects previously described in 6-week-old rats in the same model [24]. In contrast, some studies have shown increased insulin secretion due to an HP diet [45] and have even suggested that an HP diet could be beneficial in case of type 2 diabetes [46]. The increased blood glucose in the present study could be a transient effect, due to the lower carbohydrate content of the HP diet; the pups may not have been able to manage the glucose dose of the OGTT. In addition, gene expression of LPK and GK in pups exposed to the post-weaning HP diet indicate a lower hepatic glycolysis rate. The increased gene expression of PEPCK suggests a higher hepatic gluconeogenesis rate. They are all potentially related to the diet’s lower carbohydrate content. At the age of 10 weeks, the HP diet did not affect IGF-1 plasma concentration, unlike findings in 6-week-old rat pups [24], which could be an age-related effect as the animals are adult and no longer in a developmental phase. It has been proposed that increased GLP-1 plasma levels play a role in HP diet-induced satiety [47]. In contrast to a previous study, gene expression in the hypothalamus was not significantly affected by the HP diet after weaning, nor did it explain the decreased food intake [48].

The increased blood glucose concentration during the OGTT due to the post-weaning HP diet was enhanced by the HP diet during gestation. Fetal programming of glucose intolerance and diabetes has been confirmed in models of maternal protein restriction, caloric restriction, overnutrition and diabetes [49], but less extensively studied in association with protein excess during gestation. A study showed no effect on adult pup glucose metabolism [23]. However, the present study demonstrated that a post-weaning adult diet challenge, through the W diet, revealed potential detrimental effects of diet during gestation. In addition, a previous study also showed glucose homeostasis perturbation with a decreased liver insulin signaling in rat female offspring at 15-week-old [20]. These programming effects showing an impact on glucose metabolism could be a consequence of the adaptation of the fetus to the in utero nutritional environment [50] of high protein and low carbohydrates, which has been shown to increase fetal gluconeogenesis [16]. The absence of differences in insulin secretion between groups, despite a difference of glucose concentration, could represent an early sign of impaired insulin secretion and glucose intolerance.

## 5. Conclusions

The present study demonstrates that effects of an HP diet during gestation on obesity and glucose homeostasis are comparable to the effect of protein deficiency during gestation, indicating a U-shaped curve of dietary proteins during gestation plotted against the metabolic health risk of the offspring. The results reported in this work show that an HP diet given to dams during gestation, but not during lactation, in combination with a dietary challenge after weaning, increases the risk of obesity through higher fat storage capacity in adipose tissue, and can also lead to glucose intolerance in the female offspring at adulthood despite the study duration. However, the consequences on the offspring may be more highlighted in a very long-term experiment but this study contributes to valuable work previously invested on the consequences of protein excess during fetal life [20,24], also addressed in human cohorts [15]. Early life exposure to maternal nutrition can lead to epigenetic modifications revealed during adulthood through metabolic and physiological changes inducing chronic disorders such as obesity [51,52]. Epigenetic mechanisms play a key role in regulating tissue specific gene expression [2,53]. Several studies examined the relationship between maternal protein diet and epigenetic modifications. These investigations allowed to identify DNA methylations or histone modifications, targeting notably gene promoters, in different organs (liver, placenta, kidney and pancreas) [54]. Epigenetic modifications can be incriminated in the gestation effect observed in these results. Further studies could test this hypothesis.

## Figures and Tables

**Table 1:** Body adiposity and liver triglycerides at PND70, plasma lipids and glucose metabolism parameters of fasted female rat pups at PND68.

Mother’s Diet	Control			HPlact			HPgest			Gestation Effect	Lactation Effect	Post-Weaning Effect	Interactions
Pup’s Diet	NP	HP	W	NP	HP	W	NP	HP	W				
Body adiposity													
Total adipose tissue, g	28.2 ± 2.3	19.4 ± 1.3	30.2 ± 3.3	31.7 ± 3.8	17.6 ± 1.5	28.6 ± 2.6	31.5 ± 4.4	19.5 ± 1.3	42.5 ± 6.1	0.19	0.85	<0.0001	G × W 0.04
Visceral adipose tissue, g	21.3 ± 1.9	12.7 ± 1.0	22.7 ± 2.9	23.0 ± 2.9	11.8 ± 1.0	20.6 ± 2.0	20.5 ± 2.5	11.2 ± 0.8	30.0 ± 3.6	0.51	0.95	<0.0001	G × W 0.03
Subcutaneous adipose tissue, g	6.9 ± 0.7	6.7 ± 0.6	7.5 ± 0.7	8.6 ± 1.1	5.8 ± 0.7	7.9 ± 0.7	11.0 ± 2.1	8.3 ± 0.9	13.5 ± 2.8	0.04	0.76	0.005	ø
Liver lipids													
Triglycerides, g/100 g of liver	2.41 ± 0.20	2.13 ± 0.16	4.64 ± 0.83	1.88 ± 0.18	1.90 ± 0.12	3.73 ± 0.37	2.25 ± 0.24	1.96 ± 0.12	4.12 ± 0.44	0.44	0.14	<0.0001	ø
Plasma lipids													
Triglycerides, mmol/L	0.80 ± 0.10	0.71 ± 0.07	1.02 ± 0.08	0.87 ± 0.14	0.59 ± 0.04	0.69 ± 0.05	0.92 ± 0.10	0.72 ± 0.12	1.11 ± 0.13	0.36	0.31	0.03	ø
Total cholesterol, mmol/L	2.22 ± 0.18	2.32 ± 0.13	2.16 ± 0.11	2.27 ± 0.09	2.49 ± 0.37	2.06 ± 0.10	2.32 ± 0.11	2.92 ± 0.45	2.14 ± 0.10	0.25	0.87	0.01	ø
HDL cholesterol, mmol/L	1.16 ± 0.09	1.28 ± 0.07	1.12 ± 0.05	1.19 ± 0.05	1.29 ± 0.15	1.09 ± 0.05	1.25 ± 0.06	1.57 ± 0.23	1.11 ± 0.07	0.21	0.98	0.007	ø
Ratio HDL/total cholesterol	0.52 ± 0.01	0.55 ± 0.01	0.52 ± 0.01	0.53 ± 0.01	0.53 ± 0.03	0.53 ± 0.02	0.54 ± 0.01	0.54 ± 0.03	0.52 ± 0.03	0.96	0.84	0.69	ø
Nonesterified fatty acid, mmol/L	1.21 ± 0.10	1.11 ± 0.16	1.62 ± 0.13	1.33 ± 0.11	1.13 ± 0.07	1.16 ± 0.13	1.07 ± 0.11	0.91 ± 0.14	1.18 ± 0.10	0.04	0.37	0.27	ø
Glucose metabolism													
Fasted blood glucose, mg/dL	83 ± 4	95 ± 4	77 ± 3	83 ± 4	90 ± 4	82 ± 4	77 ± 3	105 ± 5	83 ± 2	0.30	0.95	<0.0001	G × W 0.02
Fasted plasma insulin, µg/L	0.18 ± 0.02	0.21 ± 0.02	0.18 ± 0.03	0.24 ± 0.05	0.14 ± 0.03	0.22 ± 0.02	0.23 ± 0.04	0.28 ± 0.06	0.21 ± 0.04	0.25	0.90	0.74	ø
HOMA-IR	0.81 ± 0.12	1.12 ± 0.15	0.76 ± 0.16	0.12 ± 0.30	0.71 ± 0.15	0.98 ± 0.14	0.98 ± 0.21	1.58 ± 0.35	0.94 ± 0.21	0.21	0.90	0.64	ø
Fasted plasma glucagon, pg/mL	30.9 ± 3.0	35.5 ± 4.4	28.0 ± 3.0	24.2 ± 4.5	25.5 ± 2.9	24.9 ± 3.0	50.9 ± 10.3	42.8 ± 8.9	44.9 ± 9.6	0.02	0.29	0.81	ø

Values are presented as means ± SEM, (*n* = 8). Effects of diets and interactions were tested within mixed model 1. G × W: gestation × post-weaning, NEFA: non esterified fatty acids, PND: post-natal day.

**Table 2:** Changes in mRNA encoding for genes in liver of fed female rat pups at PND70.

Mother’s Diet	Control			HPlact			HPgest			Gestation Effect	Lactation Effect	Post Weaning Effect
Pup’s Diet	NP	HP	W	NP	HP	W	NP	HP	W			
Liver mRNA Encoding for												
FAS	1.00 ± 0.20	0.57 ± 0.16	0.37 ± 0.09	1.19 ± 0.24	0.40 ± 0.06	0.28 ± 0.06	1.93 ± 0.35	1.21 ± 0.25	0.85 ± 0.23	0.002	0.93	<0.0001
ACC	1.00 ± 0.16	0.50 ± 0.12	0.44 ± 0.06	0.96 ± 0.21	0.46 ± 0.10	0.31 ± 0.05	1.43 ± 0.19	0.94 ± 0.23	0.81 ± 0.20	0.006	0.58	<0.0001
Scd1	1.00 ± 0.31	0.50 ± 0.14	0.17 ± 0.03	1.09 ± 0.32	0.42 ± 0.11	0.13 ± 0.03	1.64 ± 0.21	1.08 ± 0.31	0.64 ± 0.16	0.006	0.95	0.0001
MTTP	1.00 ± 0.08	0.95 ± 0.13	1.14 ± 0.22	0.99 ± 0.14	0.71 ± 0.11	0.89 ± 0.08	1.14 ± 0.11	1.20 ± 0.22	1.08 ± 0.14	0.51	0.31	0.77
GPAT	1.00 ± 0.16	0.78 ± 0.14	0.59 ± 0.10	0.97 ± 0.18	0.47 ± 0.07	0.50 ± 0.06	1.20 ± 0.17	0.95 ± 0.18	0.82 ± 0.13	0.11	0.26	0.007
DGAT	1.00 ± 0.11	1.18 ± 0.18	1.09 ± 0.24	0.99 ± 0.14	0.75 ± 0.14	0.79 ± 0.07	1.06 ± 0.09	1.50 ± 0.27	1.04 ± 0.13	0.55	0.15	0.41
CPT1	1.00 ± 0.28	1.08 ± 0.58	1.32 ± 0.46	0.42 ± 0.08	0.26 ± 0.03	0.76 ± 0.10	0.71 ± 0.13	1.23 ± 0.53	1.44 ± 0.21	0.86	0.12	0.17
ACOX1	1.00 ± 0.14	1.12 ± 0.17	1.03 ± 0.21	0.95 ± 0.13	0.74 ± 0.18	0.91 ± 0.10	0.99 ± 0.09	1.64 ± 0.36	1.23 ± 0.14	0.22	0.35	0.39
LPK	1.00 ± 0.12	0.44 ± 0.10	0.64 ± 0.14	0.86 ± 0.12	0.30 ± 0.06	0.44 ± 0.08	1.22 ± 0.15	0.74 ± 0.17	0.76 ± 0.11	0.049	0.13	<0.0001
GK	1.00 ± 0.20	0.42 ± 0.08	1.05 ± 0.23	1.26 ± 0.34	0.33 ± 0.06	0.62 ± 0.15	1.17 ± 0.32	0.58 ± 0.16	1.05 ± 0.27	0.56	0.64	0.001
PEPCK	1.00 ± 0.35	1.53 ± 0.30	1.18 ± 0.71	0.29 ± 0.11	0.87 ± 0.33	0.74 ± 0.13	0.52 ± 0.20	2.23 ± 0.66	1.02 ± 0.23	0.93	0.19	0.007
G6PC1	1.00 ± 0.17	0.42 ± 0.14	0.93 ± 0.37	0.50 ± 0.09	0.36 ± 0.07	0.55 ± 0.09	0.75 ± 0.17	1.20 ± 0.46	0.95 ± 0.17	0.55	0.26	0.72

Values are presented as means ± SEM, (*n* = 8). Effects of diets and interactions were tested within mixed Model 1. Ribosomal 18S RNA was used as the internal control. Results are expressed as ratio of expression with Control-NP group. ACC: acetyl-CoA carboxylase, Acox1: acyl-CoA oxidase 1, CPT1: Carnitine palmitoyltransferase I, DGAT: diglyceride acyltransferase, FAS: fatty acid synthase, GK: glucokinase, GPAT: glycerol-3-phosphate acyltransferase, G6PC1: Glucose-6-phosphatase, LPK: L-pyruvate kinase, LPL: lipoprotein lipase, MTTP: microsomal triglyceride transfer protein, PND: post-natal day, PEPCK: Phosphonolpyruvate carboxykinase, Scd1: stearoyl-CoA desaturase 1.

## Supplementary Materials

The following are available online at https://www.mdpi.com/2072-6643/12/2/315/s1, Table S1: Diet composition, Table S2: Primers, Table S3: Body weight and body composition at PND21. Adiposity is presented in absolute weight (g) and percentage of body weight (%), Table S4: Fasted plasma parameters of female rat pups at PND68, Table S5: Changes in mRNA encoding for genes in adipose tissue of fed female rat pups at PND70, Table S6: Changes in mRNA encoding for genes in hypothalamus of fed female rat pups at PND70. Ribosomal 18S RNA was used as the internal control. The results are expressed as ratio of expression with Control-NP group, Figure S1: Cumulative food intakes of mothers during the last week of gestation (a) and of mothers with their litter during lactation (PND0 to PND19) (b), Figure S2: Insulin area under curve during the OGTT at PND68.
