# Supplementary material for: Increased Susceptibility to Obesity and Glucose Intolerance in Adult Female Rats Programmed by High-Protein Diet during Gestation, But Not during Lactation
Source: Nutrients. 2020 Jan 25;12(2):315. doi: 10.3390/nu12020315 (PMC7071251; doi:10.3390/nu12020315)

Supplementary Materials

Table S1. Diet composition

	NP diet	HP diet	W diet
**Ingredients (g/kg)**			
Cows milk proteins	200	530	250
Cornstarch	570	287	213,9
Sucrose	92,7	45,7	213,9
Soybean oil	40	40	10
Lard	0	0	215
Mineral mix (AIN-93G-MX)	35	35	35
Vitamin mix (AIN-93-VX)	10	10	10
Cellulose	50	50	50
Choline	2,3	2,3	2,3
**Composition**			
Metabolizable energy (kCal/g)	3,5	3,5	4,5
Proteins (% energy)	20	55	20
Carbohydrates (% energy)	70	35	38
Lipids (% energy)	10	10	42

Supplementary Materials

Table S2. Primers

GENE	*FORWARD PRIMER (5′ to 3′)*	*REVERSE PRIMER (3′ to 5′)*
**In liver and adipose tissue**		
18S	ACGGAAGGGCACCACCAGGAG	GCACCACCACCCACGGAAACG
PPARɣ	TCGGATCCACAAAAAGAGTAGAAA	AACCTGATGGCATTGTGAGACA
IGF1-r	AGGAGTGTCCATCAGGCTTCA	TTCATCGCCGCAGACCTT
FAS	TGCTCCCAGCTGCAGGC	GCCCGGTAGCTCTGGGTGTA
ACC	TGGTGCAGAGGTACCGAAGTG	CGTAGTGGCCGTTCTGAAACT
Scd1	TCAGCGCTGGGAAAGTGAA	GTGTAGGAACTGGAGATCTCTTGGA
LPL	GGACTGAGGATGGCAAGCA	GGCAGGGTGAAGGGAATGTT
MTTP	TCCTCTGTTTCTTCTCCTCGTATTC	CGGTGGAGTACGTGAGCTTGT
DGAT1	GCTACGGCGGGTTCTTGA	GGGCTTCATGGAGTTCTGGAT
GPAT	CTGTATCTGCTCCCCTCTCTTTG	CGCGTTTCTCGACTCATTACTG
CTP1a	AGTTCATCCGGTTCAAGAATGG	TCACACCCACCACCACGAT
GK	TTGAGACCCGTTTCGTGTCA	AGGGTCGAAGCCCCAGAGT
L-PK	TGATGATTGGACGCTGCAA	GAGTTGGTCGAGCCTTAGTGATC
PEPCK	GGAAAGTTGAATGTGTGGGTG-AT	TTCTGGGTTGATGGCCCTTA
G6PC1	GTTCCCCGTCACCTGTGAGA	GATAGCGAGAGTAGAAGTAACCATAACG
ACOX1	AAGAAATCCCCACTGAACAAAACA	CCCAGGGAAACTTCAAAGCTT
**In hypothalamus**		
18S	GGGAGCCTGAGAAACGGC	GGGTCGGGAGTGGGTAATTT
NPY	TTTTCCTAGTTTCCCCCCACAT	CCTGGTGGTGGCATGCAT
AgRP	TGGTGCCCTTGACCAAAGTT	AATTTCTGCCCCCACAGATG
POMC	AGGCCTTTCCCCTAGAGTTCAA	GTCGGCCTTCTCGGTATCC
CART	CCGAGCCCTGGACATCTACTC	AAATACTGACCAGCTCCTTCTCATG
MC4R	TAGCCTGGCTGTGGCAGAT	CGATGGTTTCCGACCCATT
Y2R	CCGCTCCTGCTTCTGATCTC	ACCCAAAGCAGGTCCGATT
Y5R	AACCTTTGGCTCAGCATTGC	CAGAGGGCCATGACTCAACA
CRF	CAACCTCAGCCGGTTCTGA	CCCCAGGCGGAGGAAGTA

ACC: Acetyl-coa carboxylase; Acox1: acyl-CoA oxidase 1; AgRP: agouti-related protein; CART: cocaine- and amphetamine-regulated transcript; CPT1a: Carnitine Palmitoyltransferase 1; CRF: corticotropin-releasing factor; DGAT1: Diglyceride acyltransferase 1; FAS: Fatty acid synthase; G6PC1: Glucose-6-phosphatase; GK: Glucokinase; GPAT: Glycerol-3-Phosphate acyltransferase; HMGcoa: 3-Hydroxy-3-méthylglutaryl-coenzyme A; IGF1-r: Insulin growth factor 1 receptor; L-PK: L-Pyruvate kinase; LPL: Lipopotein lipase; MC4-R: melanocortin 4 receptor; MTTP: Microsomal triglyceride trasfer protein; PEPCK: Phosphonolpyruvate carboxykinase; POMC: pro-opiomelanocortin; PPARɣ: Peroxysome ploliferator-actived receptor gamma; Scd1: Stearoyl coenzym-a desaturase; Y2R: neuropeptide Y 2 receptor; Y5R: neuropeptide Y 5 receptor

Supplementary Materials

Table S3. Body weight and body composition at PND21. Adiposity is presented in absolute weight (g) and percentage of body weight (%)

Mother's diet		Control	HPlact	HPgest	Gestation effect	Lactation effect
Weight at PND21, g		49.2[a] ± 0.7	40.1[b] ± 0.7	34.9[c] ± 1.0	<0.0001	0.0003
Total adipose tissue	g	6.5[a] ± 0.2	3.9[b] ± 0.1	4.6[b] ± 0.3	0.004	0.0004
	%	12.3[a] ± 0.3	8.9[b] ± 0.2	12.3[a] ± 0.3	NS	0.0006
Subcutaneous adipose tissue	g	5.3[a] ± 0.2	3.2[b] ± 0.1	3.9[b] ± 0.2	0.01	0.0006
	%	10.2[a] ± 0.3	7.2[b] ± 0.2	10.7[a] ± 0.3	NS	0.0005
Visceral adipose tissue	g	1.15[a] ± 0.04	0.73[b] ± 0.03	0.64[b] ± 0.04	0.0002	0.002
	%	2.2[a] ± 0.07	1.66[b] ± 0.06	1.68[b] ± 0.06	0.02	0.03

Values are presented as means ± SEM, n=24, effects of diets and interactions were tested within mixed model 1. Means with different letters are significantly different (after a Tukey correction). PND: post-natal day.

Supplementary Materials

Table S4. Fasted plasma parameters of female rat pups at PND68

Mother's diet	Control			HPlact			HPgest			Gestation effect	Lactation effect	Post-weaning effect	Interactions
Pup's diet	NP	HP	W	NP	HP	W	NP	HP	W				
**Plasmatic parameters**													
Urea, mmol/L	6.19 ± 0.27	7.01 ± 0.46	5.83 ± 0.27	6.33 ± 0.47	7.58 ± 1.16	6.10 ± 0.84	6.69 ± 0.45	7.51 ± 0.83	5.65 ± 0.50	0.61	0.60	0.04	ø
Hydroxybutyrate, mmol/L	0.56 ± 0.08	0.34 ± 0.05	0.93 ± 0.06	0.68 ± 0.08	0.41 ± 0.04	0.82 ± 0.10	0.77 ± 0.07	0.44 ± 0.07	0.91 ± 0.08	0.14	0.65	<0.0001	ø
Leptin, pg/mL	3828 ± 651	2100 ± 223	3474 ± 515	4910 ± 787	1968 ± 283	4496 ± 705	5371 ± 972	2210 ± 290	5670 ± 1115	0.11	0.44	<0.0001	ø
Ratio leptin/adiposity, pg/mL/%body weight	310 ± 58	213 ± 20	267 ± 29	352 ± 47	244 ± 39	323 ± 38	338 ± 27	215 ± 31	345 ± 55	0.39	0.31	0.008	ø
IGF-1, pg/mL	1041 ± 80	829 ± 73	896 ± 63	962 ± 61	1049 ± 115	891 ± 133	745 ± 156	959 ± 111	844 ± 128	0.46	0.71	0.34	ø
PYY, pg/mL	238 ± 61	232 ± 37	173 ± 27	201 ± 26	227 ± 30	160 ± 20	247 ± 72	200 ± 42	161 ± 25	0.75	0.62	0.26	ø
GLP-1,pg/mL	85 ± 12	114 ± 21	58 ± 18	85 ± 18	125 ± 28	69 ± 10	77 ± 16	118 ± 40	99 ± 29	0.56	0.78	**0.03**	ø

Values are presented as means ± SEM, n=8, effects of diets and interactions were tested within mixed model 1. G x W: gestation x post-weaning, GLP-1: glucagon-like peptide 2, PND: post-natal day, PYY: peptide YY.

Supplementary Materials

Table S5. Changes in mRNA encoding for genes in adipose tissue of fed female rat pups at PND70

Mother's diet	Control			HPlact			HPgest			Gestation effect	Lactation effect	Post weaning effect
Pup's diet	NP	HP	W	NP	HP	W	NP	HP	W			
**Adipose tissue mRNA encoding for**												
FAS	1.00 ± 0.17	0.86 ± 0.26	0.17 ± 0.03	0.57 ± 0.05	1.72 ± 0.63	0.23 ± 0.05	1.06 ± 0.30	1.07 ± 0.23	0.45 ± 0.11	0.58	0.52	**0.0002**
ACC	1.00 ± 0.16	0.78 ± 0.26	0.15 ± 0.03	0.60 ± 0.11	1.38 ± 0.46	0.12 ± 0.02	0.86 ± 0.18	0.74 ± 0.12	0.30 ± 0.10	0.9	0.69	**0.0001**
LPL	1.00 ± 0.15	0.79 ± 0.14	0.58 ± 0.11	0.87 ± 0.20	0.97 ± 0.25	0.57 ± 0.07	1.03 ± 0.11	0.92 ± 0.20	0.94 ± 0.21	0.38	0.85	0.18
PPARγ	1.00 ± 0.19	0.64 ± 0.11	0.52 ± 0.09	1.25 ± 0.48	0.84 ± 0.17	0.57 ± 0.10	0.72 ± 0.11	1.00 ± 0.16	0.59 ± 0.11	0.79	0.39	0.11
IGF-1r	1.00 ± 0.28	0.76 ± 0.12	0.71 ± 0.13	1.13 ± 0.27	0.86 ± 0.19	0.68 ± 0.18	0.72 ± 0.14	1.22 ± 0.32	1.08 ± 0.30	0.33	0.7	0.73

Values are presented as means ± SEM, n=8, effects of diets and interactions were tested within mixed model 1. Ribosomal 18S RNA was used as the internal control. Results are expressed as ratio of expression with Control-NP group. ACC: acetyl-CoA carboxylase, FAS: fatty acid synthase, LPL: lipoprotein lipase, PND: post-natal day, PPARγ: peroxisome proliferator-activated receptor γ

Supplementary Materials

Table S6. Changes in mRNA encoding for genes in hypothalamus of fed female rat pups at PND70. Ribosomal 18S RNA was used as the internal control. Results are expressed as ratio of expression with Control-NP group

Mother's diet	Control			HPlact			HPgest			Gestation effect	Lactation effect	Post weaning effect	Interactions
Pup's diet	NP	HP	W	NP	HP	W	NP	HP	W				
**Hyothalamus gene expression**													
POMC	1.00 ± 0.13	1.06 ± 0.20	1.48 ± 0.27	0.66 ± 0.16	0.97 ± 0.15	0.52 ± 0.18	1.31 ± 0.10	1.38 ± 0.31	1.20 ± 0.21	0.65	**0.03**	0.25	ø
CART	1.00 ± 0.18	1.31 ± 0.26	1.69 ± 0.19	0.91 ± 0.19	0.91 ± 0.10	0.66 ± 0.17	1.71 ± 0.23	1.46 ± 0.17	1.44 ± 0.21	0.37	**0.023**	0.32	G x PW 0.02 & L x PW 0.03
MC4-R	1.00 ± 0.15	0.89 ± 0.11	1.19 ± 0.08	0.80 ± 0.20	0.97 ± 0.15	0.61 ± 0.19	1.89 ± 0.74	1.11 ± 0.14	1.22 ± 0.19	0.11	0.31	0.28	ø
CRF	1.00 ± 0.17	0.90 ± 0.15	1.03 ± 0.08	0.70 ± 0.17	0.94 ± 0.12	0.65 ± 0.18	1.33 ± 0.12	1.23 ± 0.19	1.39 ± 0.14	**0.02**	0.13	0.86	ø
NPY	1.00 ± 0.21	1.15 ± 0.18	1.18 ± 0.16	0.71 ± 0.11	1.07 ± 0.15	0.60 ± 0.14	1.24 ± 0.16	1.28 ± 0.11	0.98 ± 0.11	0.79	0.06	**0.03**	ø
AgRP	1.00 ± 0.16	1.01 ± 0.14	1.28 ± 0.14	2.25 ± 0.64	1.02 ± 0.20	2.14 ± 1.03	4.54 ± 3.46	1.19 ± 0.11	0.96 ± 0.14	0.29	0.51	0.17	ø
Y2R	1.00 ± 0.15	0.98 ± 0.14	1.26 ± 0.12	1.04 ± 0.19	1.03 ± 0.18	0.81 ± 0.20	1.70 ± 0.44	1.13 ± 0.09	1.24 ± 0.15	0.13	0.49	0.23	ø
Y5R	1.00 ± 0.20	0.78 ± 0.11	0.90 ± 0.15	0.80 ± 0.17	0.74 ± 0.18	0.42 ± 0.19	0.95 ± 0.11	1.15 ± 0.06	0.96 ± 0.12	0.64	0.14	0.25	ø

Values are presented as means ± SEM, n=8, effects of diets and interactions were tested within mixed model 1. Ribosomal 18S RNA was used as the internal control. Results are expressed as ratio of expression with Control-NP group. AgRP: agouti-related protein, CART: cocaine- and amphetamine-regulated transcript, CRF: corticotropin-releasing factor, G x PW: gestation x post-weaning, L x PW: lactation x post-weaning, MC4-R: melanocortin 4 receptor, NPY: neuropeptide Y, PND: post-natal day, POMC: pro-opiomelanocortin, Y2R: neuropeptide Y 2 receptor, Y5R: neuropeptide Y 5 receptor.

Supplementary Materials

Figure S1: Cumulative food intakes of mothers during the last week of gestation (a) and of mothers with their litter during lactation (PND0 to PND19) (b)

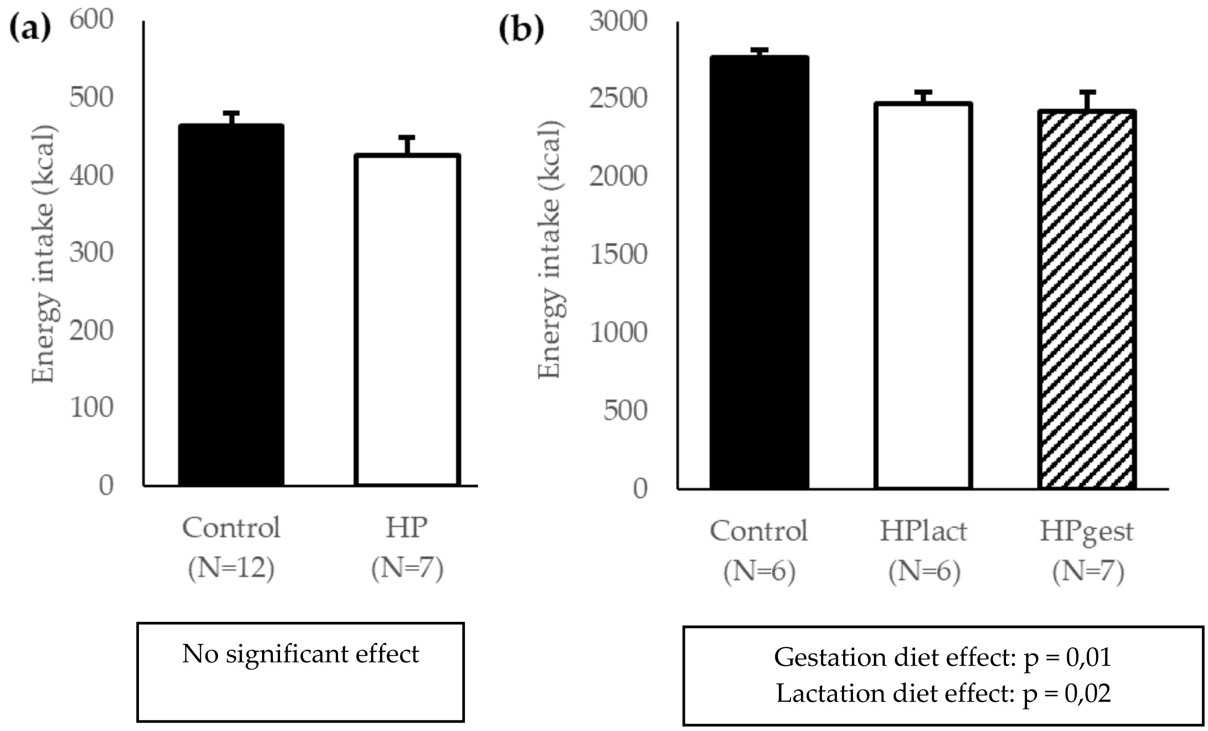

Figure S2. Insulin area under curve during the OGTT at PND68.

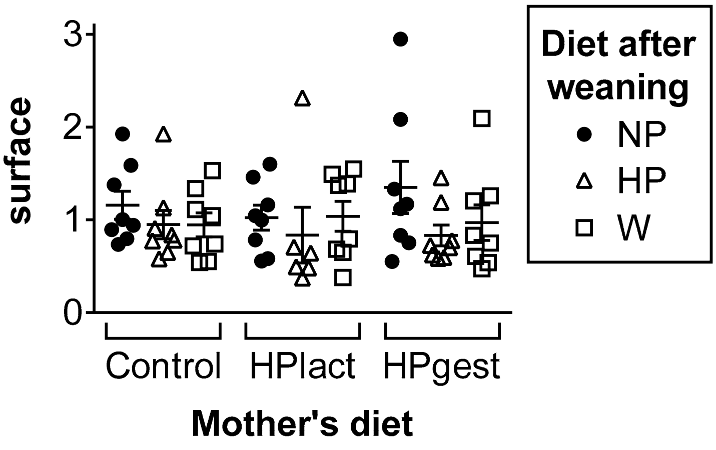

Supplement: Supplementary file 1 [file nutrients-12-00315-s001.pdf]
